# Economic evaluation of a patient and carer centred system of longer-term stroke care from a cluster randomised trial (the LoTS care trial)

**DOI:** 10.1186/1472-6963-14-S2-P92

**Published:** 2014-07-07

**Authors:** Anita Patel, Anne Forster, John Young, Jane Nixon, Katie Chapman, Martin Knapp, Kirste Mellish, Ivana Holloway, Amanda Farrin

**Affiliations:** 1 Institute of Psychiatry, King’s College London, London, UK; 2 Faculty of Medicine & Health, University of Leeds, Leeds, UK; 3 Academic Unit of Elderly Care and Rehabilitation, Bradford Teaching Hospitals NHS Foundation Trust, Bradford, UK; 4 Personal Social Services Research Unit, London School of Economics & Political Science, London, UK

## Background

Stroke generates considerable personal and financial burdens to society. We evaluated the cost-effectiveness of a new post-discharge system of care for stroke care co-ordinators (SCCs) to address the longer term problems experienced by stroke patients and their carers.

## Materials and methods

A pragmatic cluster, randomised, controlled trial compared the system of care against usual care. Randomisation was at the level of stroke service. Participants’ use of health/social care services and informal care were measured by self-complete questionnaires at baseline, 6 and 12 months. From these, we estimated and compared individual-level total costs from health/social care and societal perspectives at 6 months, 12 months and over 1 year. Costs were combined with the primary outcome, psychological health (General Health Questionnaire 12; GHQ12), and quality-adjusted life years (QALYs; based on the EQ-5D) to examine cost-effectiveness at 6 months. Cost-effectiveness acceptability curves based on the net benefit approach and bootstrapping techniques were used to estimate the probability of cost-effectiveness.

## Results

32 services were randomised, of which 29 participated, and 800 stroke patients (401 intervention, 399 control) and 208 carers (108 intervention, 100 control) were recruited. Costs of SCC inputs (mean difference £42; 95% CI: -30, 116) and total health and social care costs at 6 months, 12 months and over 1 year were similar between groups. Total costs from the societal perspective were higher in the intervention group due to greater use of informal care (+£1163 at 6 months, 95% CI 56 to 3271; +£4135 at 12 months, 95% CI 618 to 7652). There were no differences in GHQ12 or QALYs and the probability of the system of care being cost-effective at 6 months was low at the current policy threshold of £20,000 to £30,000 per QALY gain.

## Conclusions

The system of care was not cost-effective compared with usual care in this patient group over the period we examined. It is unclear why the intervention group accessed greater levels of informal care.

